# Identification positively affects the creativity of family business: The mediating role of family business support

**DOI:** 10.3389/fpsyg.2022.991899

**Published:** 2022-09-29

**Authors:** Jianjia He, Jusheng Liu, Tingting Li, Liangrong Song

**Affiliations:** ^1^Business School, University of Shanghai for Science and Technology, Shanghai, China; ^2^Center for Supernetworks Research (China), Shanghai, China; ^3^Shanghai Institute of Public Diplomacy, Shanghai, China; ^4^School of Economics and Management, Shanghai University of Political Science and Law, Shanghai, China

**Keywords:** family business, identification, family business support, creativity, mediating role

## Abstract

With the arbitrariness of family business decision-making and the complexity of interests become increasingly prominent, the transformation and innovation of family business are imminent. Under the above background, *via* analysis of data from 259 valid questionnaires from more than ten family businesses in China as a sample and with the help of the SPSS and AMOS, this study explored the impacts of identification on creativity of the family business as well as the mediating role of family business support by constructing a mediating model. The results show that the employee’s identification has a positive impact on the creativity of the family business. Besides, identification has a positive impact on family business support and family business support has a partial mediating role between identification and family business creativity. Especially, the emotional support does not have a mediating role, whereas the instrumental support has a complete mediating role between identification and family business creativity.

## Introduction

Nowadays, with the change of market environment and upgradation of the new products, to adapt to the fiercely changing market environment, the role of family business will change from inheritor to innovator. Therefore, how to improve the creativity of the family business is a quite important issue. With the development and expansion of family business, the research on family business has become an important topic. In terms of the family business, its survival and development are closely related to sustainability. One of the main objectives of family businesses is to seek sustainable development over time. It pursues long-term strategies, which is also related to the desire of the family business to maintain their ultimate control over the generations. In terms of the concept of family business, it was Donnelley who offered the first definition of the family business as one which “has been closely identified with at least two generations of a family and when this link has had a mutual influence on company policy and the interests and objectives of the family” (Donnelley, 1988). It can be seen that compared with non-family businesses, family businesses have unique family, continuity, and richness (Arsić et al., 2018). In recent years, with the acceleration of globalization and the increasingly frequent knowledge and technology innovation, family businesses are facing new challenges and competitive environment. It is important to turn to specialization and internationalization to step out of domestic market boundaries. As a result, nowadays family businesses must be competitive globally while trying to accelerate their transformation and form a unique competitiveness by enhancing their creativity (Carney et al., 2017; Hennart et al., 2019). Moreover, for China and other developing countries, due to the late development of family businesses, innovation is more prominent for them than ever before during the period of inheritance and promotion (Bennedsen et al., 2015; Xu et al., 2015).

In the development of the family business, the identification plays an important role. With regard to identification, literally, it means that identity of oneself and a kind of cognition and description of the subject itself. Besides, self-identification has a creative effect on self. If people think about their identities, they always like to propose a serious questions, such as how we see ourselves, who we think we are, what role did we play? (Farmer et al., 2003). In family business, the staff may have two identities, the family and business. On the one hand, the staff consider that they belong to a family, they must pay due responsibilities for the development and inheritance of the family. On the other hand, the staff also consider that the business is the status symbol of their identities. Therefore, identification in family business is a rich concept, if the staff recognized identities, the identification can arouse the cohesiveness between employees. Once the employees do not recognize their identities, this will cause identity conflict and affect enterprise performance (Shepherd and Haynie, 2009; Schmidts, 2013). At present, around identity in family business, existing studies have discussed it from different perspectives. Pieper (2007) considered that the identification of family enterprises has a positive impact on the development and survival of enterprises. Sundaramurthy and Kreiner (2008) considered that the convergence of family and business identity will lead to a strong consistent attitude and rapid decision-making among employees. Gómez-Mejía et al. (2007) found that a long-term identification with the business can create a self-reinforcement of family values leading to an enhance self-esteem and self-concept. Besides, identification can also increase the subjective ties and create a sense of emotional involvement in the social group (Harris and Cameron, 2005). From this point of view, as a positive factor, the identification can affect the personal behavior and the enterprise performance positively.

Furthermore, family members identification more strongly with a family business than non-family owners do with a firm, who will be more willing to do something beneficial for the company (Deephouse and Jaskiewicz, 2013). In this connection, De Massis et al. (2018) analyzed the innovation strategy of MittelStand in Germany and found that in addition to being highly innovative, these enterprises also have an important feature of being controlled and managed by the family, which reflects the strong recognition of its enterprises. Besides, some studies have pointed out that the degree of identification and emotional attachment of family business owner determine the degree of their commitment to company continuity, and this commitment will also allow family business to consider the requirements of the main stakeholders (Uhlaner et al., 2007). According to stakeholder theory, we know that the company’s stakeholders include not only the trading partners, such as shareholders, creditors, and consumers, but also the company’s internal employees. In fact, due to the special nature of family businesses, the importance of stakeholders in a family company is more complicated than in a non-family business. In this regard, Tajfel and Turner (2004) believed that a person’s social group membership and group category are important parts of a person’s self-concept. When employee in a company belong to a certain group, they will establish a special emotional connection. Park et al. (2013) believed that people in Asian countries deeply affected by Confucian culture, if the organizational identification is higher, the employees expect to establish a close relationship with the organization more. In addition, it is also pointed out that when stakeholders consider family businesses can create long-term value for all stakeholders, they will recognize the business and increase their commitment (Bosse et al., 2009), trust (Ahn and Park, 2018) and respect (He and Brown, 2013) to the enterprise, and are more willing to make companies better (Déniz-Déniz et al., 2020). Therefore, the innovation and development of the family business depend not only on the family members’ identification of the enterprise, but also on the identification of the enterprise’s stakeholders. At present, more and more studies have discovered that family businesses have formed a unique identification in long-term sustainable development (Albert et al., 2015). The employees within the company are part of the stakeholders of the family business. When the employees have this sense of identification with the family business, they will in favor of the company.

Although the identification has a positive effect on the enterprise performance, does it have an impact on the creativity of family businesses? Now, this question is not clear. As is known to all, creativity of family business is an important weapon of competition in the current market environment. Enterprises with strong creativity and innovation tend to be more dynamic, and can grasp the market opportunities to win success. In terms of the creativity of family business, most of the previous studies have focused on the influence of family business inheritors’ management style or based on socio-emotional wealth (SEW) theory (García-Sánchez et al., 2021). Besides, research on family businesses is usually carried out through case study methods, which compares family businesses with other types of organizations and understands their specific mechanisms and dynamics, especially researches on the innovation of wine family businesses. Previous studies have shown that if family business inheritors have myopia behavior, that is, managers will prefer to consider the role of family not the business, and want to select a conservative strategy to maintain the inheritance of family business. Further, managers will underinvest in long-term intangible asset projects, such as R&D investment and advertising expenditures, thereby hindering the innovation investment and output. Scholars believe that R&D activities will damage the socio-emotional wealth of family businesses, that is, SEW refers to “the emotionally related value stock generated by the family’s control position in a particular company” (Berrone et al., 2012). The focus of family businesses is not only on economic performance but also lies in the preservation of socio-emotional wealth, which is the heterogeneity between family businesses and non-family businesses, so family businesses tend to reduce R&D activities and innovative behaviors (Gomez-Mejia et al., 2014). Therefore, under the special background of family business, socio-emotion and social support are important factors in the development of family business. Exploring the creativity or performance of family business should take the role of family and the family business support into account.

Considering the above, previous studies have explored the role of identification and the creativity in family business, respectively. And scholars begin to realize that the importance of identification and the creativity in family business. However, the specific influence mechanism of identification on employee creativity is not clear, additionally, there is spare research exploring the role of family business support between identification and the creativity of family business. In fact, family business support is also a quite important concept in the development of the family business (Liu et al., 2019). When the leader of a family business is willing to consider the interests of employees and support programs and ideas from outside, it is a kind of family business support. Family business support not only can promote the success of a family business, particularly when family business members have and share mutual recognition and common goals, but also can increase the well-being of the family and business (Van Auken and Werbel, 2006). To bridge the gap that the specific influence mechanism of identification on employee creativity, this study used a mediating effect model to explore the relationship among identification, family business support, and the creativity of family business.

Our research has made some contributions in theory and practice. Firstly, this research builds a relationship between identification and creativity of family business; Secondly, we confirmed that the family business support has a partial mediating role between identification and creativity of family business; Finally, this research is helpful to improve the creativity of family business from the identification, it can provide some enlightenments for enterprise culture construction, employee loyalty improvement, and the creativity of family business. Overall, this research sheds light on the identification and creativity of family business research and can provide some enlightenments for the development of family business. To explore the relationship between identification and the creativity of family business in-depth, from the perspective of identification (Brown, 2017), this paper considers family business support as a mediating factor and attempts to investigate the mechanism by which identification affects the creativity of family businesses. The specific research questions in this article are as follows:

RQ1: *What impact does identification have on the creativity of family businesses?*
RQ2: *What effect does identification have on family business support?*
RQ3: *Does family business support play a mediating role between identification and the creativity of the family business?*

The remainder of the article is as follows: “Theory and hypotheses” develops the theory and hypotheses, the questionnaire and variable measurement is explained in “Materials and methods.” “Analysis” is the process of analysis process and we discussed the results and summarized conclusion in “Discussion.”

## Theory and hypotheses

### Identification and creativity of family business

The so-called identification refers to the process by which an individual imitates others or other groups in order to reach a partial or overall agreement emotionally psychologically (Kang et al., 2020). Identification emphasizes that employees and corporate values are consistent, which is reflected in the emotional dependence of each employee on the company’s sense of belonging, pride, and loyalty (Déniz-Déniz et al., 2020). For a family business, the attention and recognition of the company’s values and vision will promote the long-term development of the company. Apart from it, when the family business has external spiritual capital, they may have a better opportunity to make innovation in the daily operation of technology and business (Duran et al., 2016). In terms of creativity of the family business, according to the definition of Im and Workman (2004) the creativity of the family business can be divided into two dimensions: novelty and feasibility. Novelty is the originality and uniqueness of creative ideas; while feasibility is the appropriateness and usefulness of creative ideas for enterprises. At present, previous studies have confirmed that the high level of identification can motivate employees to dedicate to the enterprise (Cai et al., 2019; Ma et al., 2020). This is consistent with the creative role identity theory (Farmer et al., 2003). In the role identity theory, if ones’ role identity is stronger, people will more believe that they have the ability to engage in innovative activities, and they can more actively respond to the difficulties and risks when they encountered in innovation activities (Liu et al., 2021). Finally, the creativity of the employees will be improved (Williams et al., 2021; Anglin et al., 2022).

Furthermore, the identification of the employees is stronger, it is easier to form a harmonious working atmosphere within the enterprise (Guo et al., 2020). The employees are more willing to establish a lasting relationship with the company, and combine their development with the company’s vision, thereby promoting the creativity of the family business. On the one hand, employees can help companies to obtain more resources when they have a high level of identification, and they can also develop strategic resources to support and enhance the efficiency of organizational mechanisms and processes, and enhance the flexibility of organizational resources (Déniz-Déniz et al., 2020). As a result, the creativity of the family business can be further improved. On the other hand, high-level identified employees will reach a consensus with the goals and strategies of the family business, which can also enhance the motivation of knowledge sharing within the organization, establish good communication between executives and employees, and thus affect the willingness of family business’s employees to improve the creativity of the organization. Therefore, we make the H1a and H1b as follows:

*H1a*: Identification is positively related to the novelty of creativity, that is, the stronger identification of employees, the more innovative ideas of family business are.

*H1b*: Identification is positively related to the feasibility of creativity, that is, the stronger identification of employees, the more feasible ideas of family business are.

### Identification and family business support

The business is not only a provider of employees’ economic resources, but also an important source of meeting employees’ value needs, such as affirmation of work and concern of the company. In order to specifically show the employee’s family business support, this paper divides it into two dimensions, namely emotional support and instrumental support. In a family business, when employees regard themselves as a member of the family business (Shen and Benson, 2014), they will think that the superiors and subordinates are consistent in the exchange level. At this time, the subordinate employees will have a strong sense of organizational support, which in turn will promote the development of family business.

In addition, the family company’s care will greatly enhance the employee’s sense of belonging to the company, that is, enhances their identification, thereby, further enhances the employee’s organizational support. Basly and Saunier (2020) found that the family member’s identification is stronger, their commitments to the firm and family business are more, and they have more emotional attachment with the family business, therefore, they will obtain more emotional and organizational support. Mahto et al. (2014) considered that the larger family businesses are more concerned about the staff’s identification and give them more socio-emotional wealth to maintain the staff’s continuation commitment. Cabrera-Suárez et al. (2014) also considered that the family identification can lead to the firm staff to contribute their valuable creations and achieve their goals.

Moreover, employees’ psychological emotions also play a very important role in their integration into a family business (Pieper, 2010; Ramos et al., 2014). The individual’s sense of identification is stronger, the degree of employee integration into the enterprise is higher, and it is more beneficial to reduce the psychological distance between employee and family business, ease their sense of alienation from the family business, and increase employees’ emotional support (Wielsma and Brunninge, 2019). When employees feel a sense of alienation from the company is weaker, the embedding of the family business is stronger, and the employee is more beneficial to obtain resource information, material, and other instrumental support (Zellweger et al., 2010). Based on the above description, we propose:

*H2a*: Identification is positively related to emotional support, that is, the stronger employee identification is, the higher the emotional support of family business is.

*H2b*: Identification is positively related to instrumental support, that is, the stronger employee identification is, the higher the instrumental support of family business is.

### The mediating role of family business support

The essence of organizational support is the organization’s commitment and encouragement to employees, such as fair evaluation of new ideas, encouragement of rational and innovative ideas, and recognition of creative achievements (Madjar, 2008). As an external resource, similar to organizational support, family business support plays an important role in the improvement of employees’ creativity. According to the creative role identity theory, the creativity of employees is not only related to their own role identity, but also closely related to their external environment. In this regard, Tierney and Farmer (2002) considered that the support of superiors is an important source of creativity self-efficacy, that is, social support is helpful to promote creativity. Zhang and Bartol (2010) found that the identification can affect intrinsic motivation and creative process engagement by the psychological empowerment. Shen and Benson (2014) found that organizational support can not only directly promote employee task performance and out-of-role help behavior, but also regulate the role of organizational identification in task performance and out-of-role help behavior. Besides, social exchange theory considered that interaction and exchange are two core contents in microsociology (Choi et al., 2015). Similarly, in family business, if the employees have high identification, they will have a clearer positioning of their own roles, and it is easier to form strong organizational commitment. As an exchange, the enterprise will provide some emotional support, such as the leaders of enterprise will recognize the employee’s status and give more encourage to let employees carry out more innovative activities. Further, the emotional support will enhance employees’ sense of mission to the organization, in order to repay the organization, employees will form a positive attitude such as emotional commitment, and then show high creativity or innovative behavior (Zahoor et al., 2022). Therefore, we propose following hypotheses:

*H3a*: Emotional support is positively correlated with the novelty of creativity, that is, the stronger the emotional support is, the more novel the creative ideas of the family business are.

*H3b*: Emotional support is positively correlated with the feasibility of creativity, that is, the stronger the emotional support is, the more feasible the creative ideas of the family business are.

Besides, in addition to emotional support, if the enterprise provides enough resources, it will help employees to reduce the uncertainty of innovative work, strengthen the sense of meaning of creativity, and enhance the role identity and creativity of employees (Nazir et al., 2018; Greco et al., 2022). As a kind of family business support, instrumental support also has an important status in the development of employees and construction of the enterprise culture and team building. Generally speaking, instrumental support refers to the help provided by the organization in time when employees encounter problems in the process of work, for example, the method support, creating an information exchange platform, and building a knowledge exchange platform for employees. Previous studies have confirmed that the instrumental support has a positive effect on the creativity of employees. For example, Baer et al. (2003) found that the economic rewarding can positively affect the creativity of employees. Kessel et al. (2012) considered that instrumental support can improve the team’s ability to generate new ideas. Van Knippenberg et al. (2004) hold the view that the instrumental support can provide methods for the employees to promote the exchange of views and the integration of perspectives, further improve the total creativity. Truong et al. (2021) found that employee engagement can positively affect the creativity and the coworker support from colleague can strengthen the effect of on work engagement on the creativity. On the whole, current researches believe that instrumental support has a positive impact on employees’ creativity. On the one hand, instrumental support such as information exchange and knowledge sharing can promote employees’ innovative ability and encourage employees to come up with original ideas. On the other hand, good instrumental support can help employees to improve the appropriateness, cope with various challenges, and complete the innovation activities quickly and efficiently based on the specific situation. Therefore, this paper makes the following hypotheses:

*H4a*: Instrumental support is positively related to the novelty of creativity, that is, the stronger the instrumental support is, the more novel the creative ideas of the family business are.

*H4b*: Instrumental support is positively related to the feasibility of creativity, that is, the stronger the instrumental support is, the more feasible the creative ideas of the family business are.

### Model

**Figure 1:**
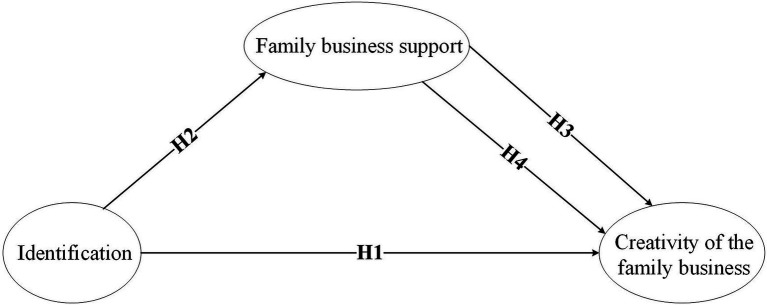
Research model.

Based on the above analysis, a relationship model of identification, family business support, and the creativity of the family business is established, as shown in Figure 1.

## Materials and methods

### Samples and data

In order to obtain accurate and effective numbers and statistical results to scientifically measure the relationship between identification and creativity of family businesses, this paper designed a survey scale. We contact family businesses on the Internet and encourage them to fill out questionnaires. The questionnaire targets more than 10 family enterprises in Zhejiang, Shanghai, Jiangsu, and Anhui provinces in China. A total of 280 questionnaires were issued, and 259 were effectively recovered (the recovery rate was 92.5%). The family companies selected in the sample are mainly in the fields of electronic information, bio-medicine, precision instruments, and automobile manufacturing, where cooperative R&D and innovation activities are frequent and products are updated quickly. Among the sample enterprises, 30% are small enterprises, 50% are medium-sized enterprises, and the remaining 20% are large enterprises; employees are mainly ordinary employees (54.3%), grassroots managers (23.7%), middle-level managers (13.6%) and senior managers (8.4%); the men surveyed accounted for 62.5% and the women accounted for 37.5%; the working age of employees in current enterprises was 22.5% below 3 years, the working years of 3 years to 5 years were43.8%, and the working years of above 5 years were 33.7%.

### Questionnaire and variable measurement

In order to ensure the scientificity of the questionnaire items, this paper conducted a pre-survey of the questionnaire to evaluate the rationality of the questionnaire design firstly, and then modified the questionnaire according to the feedback and opinions of the pre-testers. Each variable item adopts Likert’s 5-point score method. According to the score of 1 to 5 points, it represents five levels from “completely disagree” to “completely agree.” The specific questionnaire items are designed as follows:

#### Identification

At present, there are few measurement questionnaires on identification. Based on the measurement table designed and compiled by Johnson et al. (1999), this study appropriately modified scale under the research purpose and specific circumstances of this paper, and generated a summary identification scale suitable for this article. The scale includes 6 items, such as “I think the problem that the company is facing is the problem that I am facing,” “I have a strong sense of belonging in the company,” “My knowledge and skills can be fully obtained and effectively played in the company,” and “the social image of the company can represent my image very well” and so on.

#### Family business creativity

Regarding the measurement questionnaire of corporate creativity, Im and Workman (2004) believed that corporate creativity can be divided into two dimensions: novelty and feasibility. Therefore, this paper draws on the research results of Amabile et al. (1996) and combines the measurement scales of Im and Workman (2004) to measure the creativity of family businesses from six items in the two dimensions of novelty and feasibility. For example, “Company produces new ideas every year”, “The company actively creates an environment to generate new ideas”, “The company will often spend time discussing the practicality of innovative ideas”, and “The company will proactively provide a place and environment to verify the practicality of innovative ideas”.

#### Family business support

For the measurement of the mediating variable of family business support, this paper divides family business support into two dimensions: emotional support and instrumental support. To measure the family business support, we designed 10 items, such as “I can seek the company’s help when I encounter difficulties at work,” “The company pays attention to my opinions,” “The company provides me with the informational and emotional support needed for work,” “The company attaches great importance to my personal development,” and “The company provides me with all kinds of material resources needed for work.”

## Analysis

### Reliability and validity test

In order to ensure the reliability and consistency of the questionnaire measurement, this paper first carries out reliability test and validity test on the questionnaire scale. We use SPSS22.0 to do the reliability test and use Cronbach’s consistency coefficient to analyze the reliability level of the measured table items. The coefficient can be divided into three categories: 0.6–0.7 means acceptable; 0.7–0.8 means good reliability; 0.8–0.9 means the data is ideal.

#### Reliability test

The coefficients of identification, family business support, and family business creativity questionnaire scale and the coefficients based on standardized items are shown in Table 1 respectively. It can be seen from Table 1 that the reliability of each variable meets the requirement of greater than 0.7. The results are ideal, and the consistency and reliability of the scale are high.

**Table 1:** Variable coefficient values.

Research variables	Cronbach-α	Based on standardized items Cronbach-α
Identification	0.823	0.823
family business support	0.898	0.898
Creativity of family business	0.884	0.884

#### Validity analysis

Before the final formal analysis, as a kind of common method variance (CMV), we use Harman’s single-factor test to examine whether there is a common systematical error caused by the measurement item. The result shows that the variance explained by the first factor does not exceed half of the total variance explained, therefore, there is no common method variance (Podsakoff and Organ, 1986; Podsakoff, 2003). Using Lisrel8.7 to conduct confirmatory factor analysis (CFA) on the main variables, the specific results are shown in Table 2. It can be seen from Table 2 that the chi-square degree of freedom ratio of the three-factor model is 2.26 and less than 3, the values of CFI, IFI, and NFI are 0.97, 0.97, and 0.94, respectively, better than other two-factor and single-factor models. In addition, the standardized factor loading corresponding to each latent variable is greater than 0.6 and has statistical significance at the level of *p* < 0.001. By calculating the AVE value of each variable on the diagonal, it is found that the value is not lower than the minimum value of 0.5, this result shows that the measurement scale has good aggregation validity. Moreover, the square root of the average variance extracted of each variable is greater than the correlation coefficient of the variable and all other variables, indicating that the measurement scale has good discriminatory validity. In summary, the effect of this paper on the measurement of the three variables of identification, family business support and family business creativity is satisfactory.

**Table 2:** Confirmatory factor analysis results.

Model	*x*^2^/*df*	RMSEA	SRMR	CFI	IFI	NFI
Three-factor model	2.26	0.065	0.045	0.97	0.97	0.94
Two-factor model a^1^	3.34	0.123	0.067	0.94	0.94	0.92
Two-factor model b^2^	3.68	0.121	0.100	0.93	0.93	0.92
Single-factor c^3^	4.53	0.194	0.130	0.87	0.87	0.86

*n* = 259; a^1^ merges identification and family business creativity into one potential factor; b^2^ combines identification and family business support as one potential factor; c^3^ attributes all projects to the same potential factor.

### Descriptive statistics and related analysis

Through SPSS22.0, the relevant analysis of the research variables was carried out to explore the interdependence between identification, family business support and family business creativity, as well as the relationship between the secondary variables, such as “industry type” and “employee title,” “enterprise size,” “working years “and” gender “, and the main variables, the specific results are shown in Table 3.

**Table 3:** Related analysis results.

Variables	Mean value	Standard deviation	1	2	3	4	5	6	7	8
Family business creativity	3.43	0.78	1							
Family business support	4.23	0.65	0.33^***^	1						
Identification	3.48	0.69	0.29^***^	0.32^***^	1					
Industry type	2.45	1.14	0.14^**^	−0.09	−0.00	1				
Employee title	2.30	0.96	0.11^*^	0.13^*^	0.01	0.11	1			
Enterprise size	2.12	1.15	0.14^**^	−0.00	0.11	0.22^**^	−0.02	1		
Working years	2.48	0.87	−0.16	0.17^**^	0.09^*^	−0.01	0.11	0.15	1	
Gender	2.43	0.51	0.07	−0.04	−0.13	0.11^*^	0.08	0.03	0.12^*^	1

^*^p < 0.05;^**^p < 0.01;^***^p < 0.001.

Table 3 shows the descriptive and correlation analysis results of the design variables. It can be seen from Table 3 that the average value of the creativity of the sample family companies is 3.43, indicating that the creativity level of the sample companies tends to be neutral. Meanwhile, this variable also has a relatively obvious standard deviation, indicating that the creativity levels of different family companies have a gap. The average value of family business support is 4.23, indicating that the sample business support exceeds the average level. Its standard deviation is 0.65, indicating that the sample company has a significant gap in family support. Further from Table 3, we can find that identification is significantly related to family business support, and identification is significantly related to the creativity of the family business, which provides the possibility of verifying the existence of mediating effects.

### Hypothetical testing

#### Identification and creativity of family business

**Figure 2:**
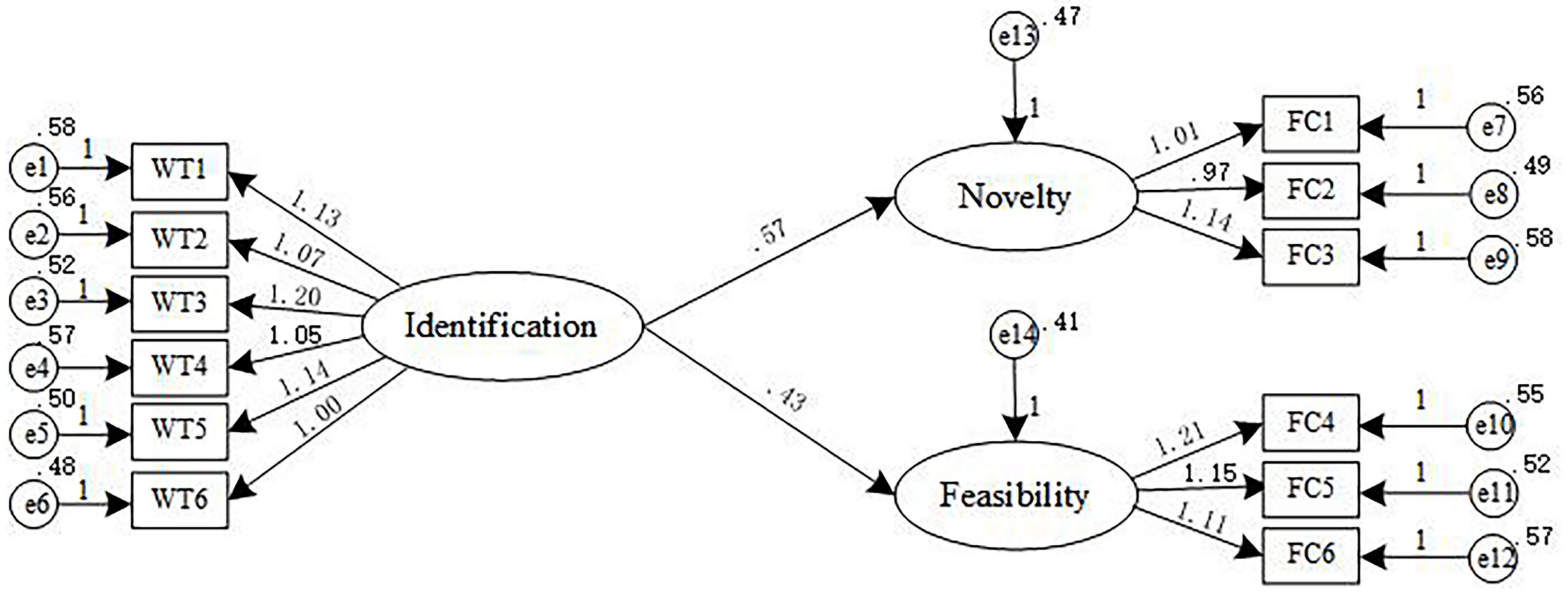
Relationship between identification and the creativity of family businesses.

In order to verify that identification has a significant impact on the creativity of family businesses, AMOS 23.0 was used to analyze the impact of identification on all dimensions of family business creativity, to visually discover the specific impact of identification on each dimension of family business creativity. The model of the effect of employee identification on the creativity of family business is shown in Figure 2. Relationship between identification and the creativity of family businesses, and the path coefficient and significance level are shown in Table 4.

**Table 4:** Path coefficient and significance level between identification and creativity of family business.

Path of action	Estimate	S.E.	C.R.	*p*
Novelty ← identification	0.567	0.080	5.116	^***^
Feasibility ← identification	0.432	0.074	5.207	^***^

^*^ *p* < 0.05; ^**^p < 0.01; ^***^p < 0.001.

It can be seen from Table 4 that the path coefficients of employee identification on the novelty and feasibility of the creativity of family business are 0.567 and 0.432, respectively, which are significantly positively correlated at the level of *p* < 0.001. Therefore, identification has a positive effect on the novelty and feasibility of the creativity of family businesses, Hypothesis1a and Hypothesis1b are established.

#### Identification and family business support

**Figure 3:**
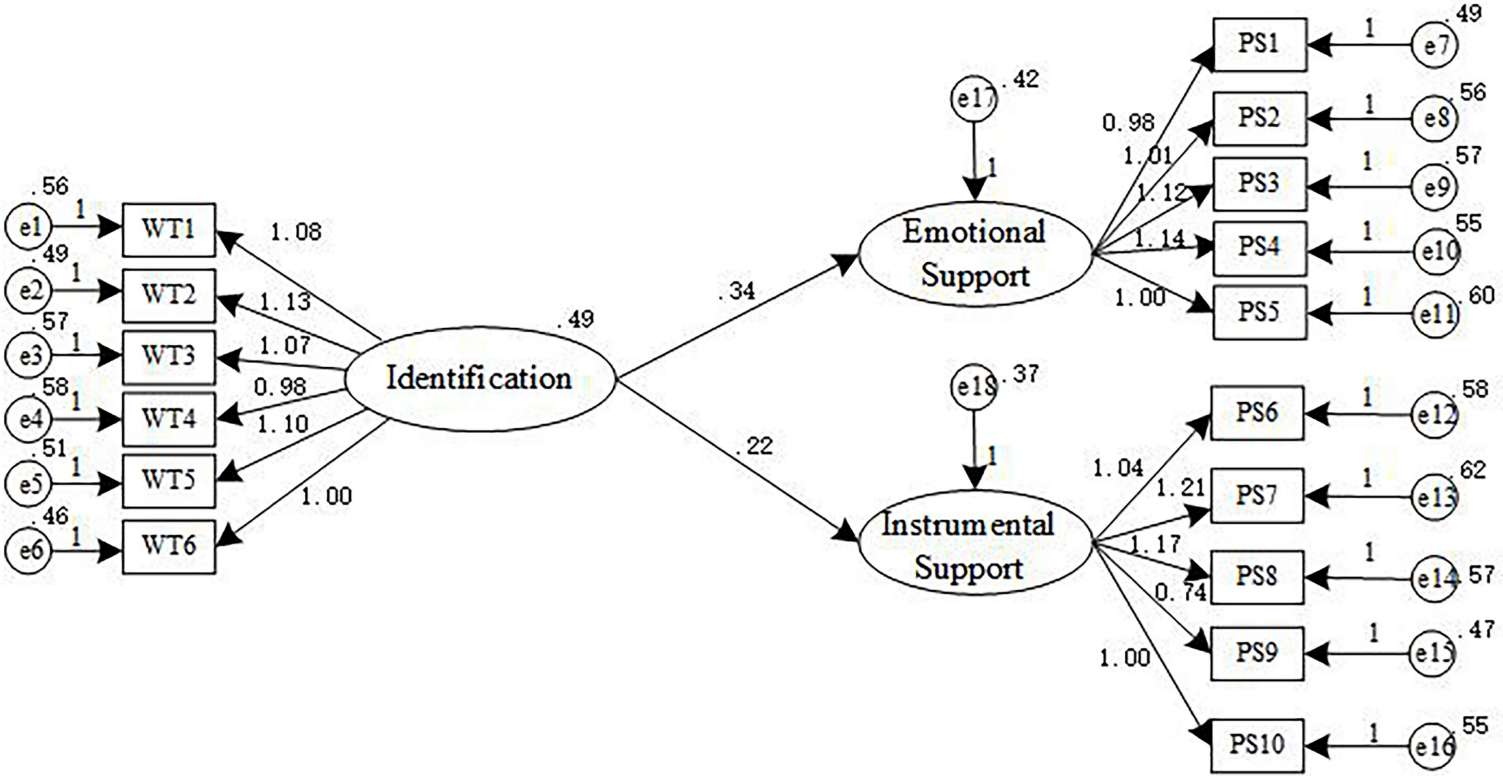
Relationship between identification and family business support.

To verify that identification has a significant impact on family business support, AMOS23 was used to analyze the impact of identification on each dimension of family business support. To find out the concrete influence of identification on each dimension of family business support. The model of the effect of employee identification on family business support is shown in Figure 3, and the path coefficient and significance level are shown in Table 5.

**Table 5:** Path coefficients and significance levels between identification and family.

Path of action	Estimate	S.E.	C.R.	*p*
Emotional support←Identification	0.336	0.057	4.256	^***^
Instrumental support←Identification	0.221	0.062	3.485	^***^

^*^p < 0.05; ^**^p < 0.01; ^***^p < 0.001.

It can be seen from Table 5 that the employee’s identification and the path coefficients of emotional support and instrumental support of the family business are 0.336 and 0.221, respectively, which are significantly positively correlated at the level of *p* < 0.001. Therefore, identification has a positive impact on the emotional support and instrumental of the family business, Hypothesis2a and Hypothesis2b are established.

#### The mediating role of family business support

**Figure 4:**
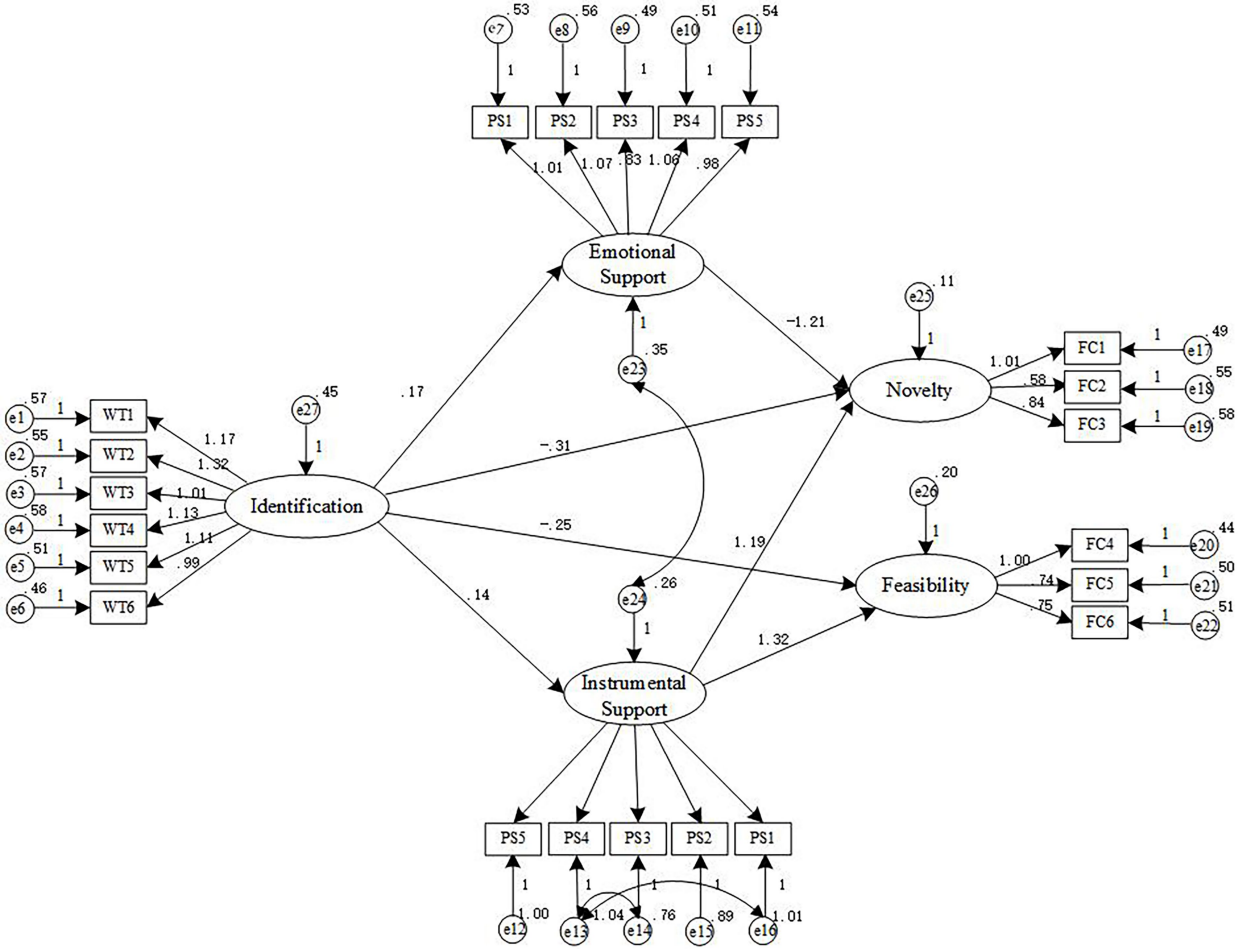
Mediating role model (after revision).

After importing the sample data, AMOS23.0 was used for calculation, and the initial structural equation model-fitting effect and mediating model fitting index were obtained, as shown in Table 6. From Table 6, the *x*^2^/*df* value of the model is 3.65, which is greater than 3. The values of TLI, IFI, NFI, are all greater than 0.7, the CFI value is greater than 0.8, and the RMSEA value is less than 0.08. It can be seen from the above data that the fitting effect of this model is general, so this model needs to be revised. To make the model fit index ideal, we increase the correlation path between the residual variables according to the value of modification index (MI). Therefore, according to MI value in the data output of AMOS23.0, the correlation paths between e13 and e14, e13 and e16, e23 and e24 residual variables are sequentially increased, and the revised model is shown in Figure 4.

**Table 6:** Intermediate model fitting index (before modification).

Index	*x*^2^/*df*	TLI	IFI	NFI	CFI	RMSEA
Model	3.650	0.775	0.797	0.742	0.811	0.076

Table 7 shows the test results of the mediating role of family business support. It can be seen from Table 7, the path coefficient between employee identification and novelty is –0.312, and the path coefficient between employee identification and feasibility is –0.250, both of which are significantly related at *p* < 0.001 level. Employee identification and the path coefficient between emotional support is 0.168, the path coefficient between instrumental support is 0.138, the path coefficient between emotional support and creativity novelty is –1.212, the path coefficient between instrumental support and creativity novelty is 1.185, and the path coefficient between instrumental support and creativity feasibility is 1.315, which are significantly correlated at least at *p* < 0.01. Therefore, Hypothesis3a and Hypothesis 3b are not supported, Hypothesis 4a and Hypothesis 4b are supported.

**Table 7:** Test results of the mediating role of family business support.

Path of action	Estimate	S.E.	C.R.	*p*
Novelty ← identification	−0.312	0.064	−3.423	^***^
Feasibility ← identification	−0.250	0.113	−3.145	^***^
Emotional support ← identification	0.168	0.053	2.117	^**^
Instrumental support ← identification	0.138	0.050	3.133	^***^
Novelty ← emotional support	−1.212	0.341	−4.257	^***^
Novelty ← instrumental support	1.185	0.202	5.412	^***^
Feasibility ← instrumental support	1.315	0.335	5.655	^***^

^*^p < 0.05; ^**^p < 0.01; ^***^p < 0.001.

To display the hypothesis test results clearly, we further present the final hypothesis test results in Table 8.

**Table 8:** Hypothesis test results.

Hypothesis	Hypothetical content	Test results
H1a	Identification is positively related to the novelty of creativity.	Supported
H1b	Identification is positively related to the feasibility of creativity.	Supported
H2a	Identification is positively related to emotional support.	Supported
H2b	Identification is positively related to instrumental support.	Supported
H3a	Emotional support is positively correlated with the novelty of creativity.	Unsupported
H3b	Emotional support is positively correlated with the feasibility of creativity.	Unsupported
H4a	Instrumental support is positively related to the novelty of creativity.	Supported
H4b	Instrumental support is positively related to the feasibility of creativity.	Supported

## Discussion

At present, many studies have explored family businesses (Hofman and Newman, 2014; Miller, 2014; Overbeke et al., 2015). Unlike previous studies, this article mainly investigates the impact of identification on the creativity of family business, and explores the mediating role of family business support between identification and creativity of family business. On the basis of relevant literature reviews and theoretical analysis, it puts forward relevant hypotheses and constructs a model of identification, family business support and the creativity of family business. Finally, the data are collected from the questionnaire survey and empirically analyzed to obtain the relevant conclusions. The specific conclusions are as follows:

Firstly, the employee’s identification has a positive impact on the creativity of the family business. In fact, previous studies have confirmed that the recognition of knowledge, skills and work achievements (Lin et al., 2016), large promotion space, harmonious relationships with colleagues (Valackienė, 2009), solidarity and mutual assistance in the corporate working atmosphere (Johnson et al., 2012) can make employees’ identification more effective and stronger (Johnson et al., 2012). Under the atmosphere of high identification, the employees can coordinate their interests with the interests of the enterprise, and promote the personal performance and job satisfaction (Lee et al., 2015), further create innovative ideas of the family business. This conclusion is also similar to most studies (Judge et al., 2001; Carmeli et al., 2011).

Secondly, identification has a positive impact on family business support. This is because on the one hand, the stronger the employee’s sense of identification, the higher the employee’s integration into the family business, which reduces the psychological distance between the employee and the company, thereby increasing the employee’s emotions such as respect, recognition, and praise from the family business (Becker et al., 1996). On the other hand, a strong sense of identification makes the goals of employees and the company reach an agreement, which also helps employees get instrumental support such as information and materials (Greco et al., 2022).

Thirdly, family business support has a partial mediating role between identification and family business creativity. Different from existing studies (Van Auken and Werbel, 2006; Klyver et al., 2018), compared with the emotional support, our research conformed that the instrumental support plays a complete mediating role between identification and the creativity of family business. On the whole, the mediating role of family business support is mainly reflected in that when employees perceive the company’s instrumental supports for themselves, they often give more feedback to the company. A strong sense of identification enables them to actively contribute to family businesses, and they are more willing to try innovative ideas, thus promoting the continuous improvement of the creativity of the family businesses (Ahmad et al., 2022).

### Theoretical implications

In theory, first of all, this research explores the impact of employee identification on the creativity of family businesses, and finds that the identification has a positive impact on the creativity of family businesses. It extends the boundary for the work of identification (Abdelmotaleb et al., 2018) and the creativity (Tong et al., 2019), and builds a relationship between identification and creativity (Szostak, 2020). It also provides a mechanism and path to improve the creativity of family business and some guidance for the research of family business creativity and identification.

Secondly, another important contribution of the current work is that it emphasizes the role of family business support in family business research. Generally speaking, family business support is a kind of social support. The social support can be divided into the emotional support and the instrumental support. From this research, we can find the instrumental support plays a complete mediating role between identification and creativity, whereas the emotional support has no mediating role. This discovery explores the role of family business support in detail and provides some theoretical enlightenment for the research of family business support.

### Practical implications

To begin with, identification is a strong psychological link to maintain the relationship between individuals and individuals, and between individuals and enterprises. When the family business is innovating, it can start from the perspective of identification, from the perspective of improving employees’ work enthusiasm, inspiring employees to display their own knowledge, skills and talents, and providing employees with fair and competitive work opportunities.

Next, in work, family businesses should increase their concern and recognition for employees, shorten the distance between the company and employees psychologically and emotionally, improve the employees’ sense of cooperative spirit, encourage and innovative ideas, and constantly encourage employees to combine their work with the long-term interest and value of the company, further, it contributes to the long-term development of enterprises.

Last but not least, the support of family business has an important role in promoting the innovation of the family business. When family business carry out activities, the company should pay more attention to the emotional care of employees, such as through certain material and spiritual incentives, so that employees can get some emotional care and their identification can be enhanced effectively. Ultimately, employees are encouraged to put forward innovative ideas for the family enterprise and help the family business to transform and upgrade.

### Limitations and future research

Although, we do a detailed study around the impact of identification the creativity of family business, there is still some limitations. Firstly our samples come from Yangtze River Delta region in China. Although the data are representative, there are still certain geographical restrictions in our data. To obtain more robust conclusion, future researches still need to obtain data in other regions, even foreign family business data.

Secondly, this study focuses on exploring the creativity of family businesses from the perspective of employees’ identification and family businesses support, and provides a perspective of hoping to arouse researchers’ attention to stakeholders of non-family members in family businesses. However, it cannot control others factors which influence the creativity of family business, such as corporate culture and atmosphere. Future researchers also can control the impact of corporate culture and the corporate atmosphere on the creativity of the family business.

Thirdly, although this study used the quantitative method and questionnaire survey to measure the employees’ identification, family businesses support, and the creativity of family businesses. However, on data acquisition, questionnaire survey may cannot reflect the participants’ opinions well. Future research can also combine the qualitative and quantitative methods to explore related research around family businesses accurately and perfectly. Besides, in terms of research content, although this research explore the identification in family business, there is less family factor in this research, the identification is more business oriented than family oriented. Therefore, future research may consider the effect of consistency and conflict of identification between family and business on the creativity in family business.

## Conclusion

To conclude, the current research explored the relationship between the identification and the creativity of family business, meanwhile, it also investigated the mediating role of family business support. This research cleared the action path from identification to the creativity of family business. Based on a mediating model, to tests the relationship among identification, family business support, and creativity of family business empirically, this study collected 259 effective questionnaires from the fields of electronic information, bio-medicine, precision instruments, and automobile manufacturing in China. After a series of tests, the research found that the identification has a positive effect in the creativity of family business. Meanwhile, the family business support has a partial mediating between the identification and the creativity of family business. The identification can influence the creativity of family business through instrumental support, whereas emotional support does not have a mediating role. This research will provide some theoretical and practical implications to the development of family business.

## Data availability statement

The datasets presented in this article are not readily available because of the requirement to protect business information. Requests to access the datasets should be directed to the corresponding author.

## Ethics statement

Ethical review and approval was not required for the study on human participants in accordance with the local legislation and institutional requirements. Written informed consent from the patients/ participants or patients/participants legal guardian/next of kin was not required to participate in this study in accordance with the national legislation and the institutional requirements.

## Author contributions

HJ provided the ideas and designed the overall model, and wrote manuscript. LJ wrote and modified manuscript. LT collected and analyzed data. SL put forward some suggestions in the process of writing and improving the manuscript.

## Funding

This work was supported by the National Natural Science Foundation Project (grant no. 71871144) and the Science and Technology Development Program of University of Shanghai for Science and Technology (grant no. 2020KJFZ046).

## Conflict of interest

The authors declare that the research was conducted in the absence of any commercial or financial relationships that could be construed as a potential conflict of interest.

## Publisher’s note

All claims expressed in this article are solely those of the authors and do not necessarily represent those of their affiliated organizations, or those of the publisher, the editors and the reviewers. Any product that may be evaluated in this article, or claim that may be made by its manufacturer, is not guaranteed or endorsed by the publisher.
